# Dynamic change of the gastrointestinal bacterial ecology in cows from birth to adulthood

**DOI:** 10.1002/mbo3.1119

**Published:** 2020-10-09

**Authors:** Chun Y. Guo, Shou K. Ji, Hui Yan, Ya J. Wang, Jing J. Liu, Zhi J. Cao, Hong J. Yang, Wen J. Zhang, Sheng L. Li

**Affiliations:** ^1^ College of Animal Science and Technology Shihezi University Shihezi China; ^2^ State Key Laboratory of Animal Nutrition Beijing Engineering Technology Research Center of Raw Milk Quality and Safety Control College of Animal Science and Technology China Agricultural University Beijing China; ^3^ Jinzhong Vocational and Technical College Jinzhong China; ^4^ College of Animal Science and Technology Hebei Agricultural University Baoding China

**Keywords:** bacterial ecology, cows, maturity, microbiota establishment

## Abstract

The gut microbiota plays multiple critical roles in maintaining the health of the host, especially in ruminants. However, our understanding of the establishment of gut microbiota from birth to adulthood is still limited. To address this, the bacterial ecology of the rumen, abomasum, duodenum, and rectum in Holstein cows ranging in age from 1 week to 5 years old was investigated using 16S rRNA gene sequencing in this study. A major change in the composition, diversity, and abundance of bacteria was observed with increased age (*p* < 0.05). Microbiota gradually matured in each gut segment and followed the Gompertz model when the Chao1, Shannon, and maturity indexes (*p* < 0.05, *r* > 0.94) were applied. Importantly, the Gompertz model parameter differed between the gut segments, with the highest microbiota growth rate found in the rectum, followed by the rumen, abomasum, and duodenum. Compared to older animals, greater microbiota similarities were found in the adjacent gut segments of younger animals (*p* < 0.05). Our findings indicate that gut microbiotas are established quickly when cows are young and then slow with age and that early in life, hindgut microbiota may be more easily affected by the foregut microbiota.

## Graphical abstract

Microbiota gradually matured followed the Gompertz model in each gut segment (rumen, abomasum, duodenum, and rectum) of cows. Microbiota in the rectum matured earliest followed by that in abomasum and rumen, and microbiota in the duodenum matured latest.

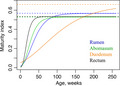

## 1 INTRODUCTION

Gut microbiota has been demonstrated to be important for host health, especially in ruminants such as cows that rely on gut fermentation to convert indigestible plant biomass into food products (Hess et al., 2011; Rubino et al., 2017; Shabat et al., 2016). Considerable research has focused on bovine intestinal microbial composition (Durso et al., 2010; Malmuthuge, Li, Goonewardene, Oba, & Guan, 2013; Uyeno, Sekiguchi, & Kamagata, 2010), compositional differences in different gut segments (Frey et al., 2010; Gu et al., 2013; Mao, Zhang, Liu, & Zhu, 2015; de Oliveira et al., 2013), and the functions of the gut bacterial community (Popova et al., 2017). These previous studies have expanded our knowledge of the gut bacterial community.

The gastrointestinal microbiota is a dynamic system that changes with host development (Dill‐McFarland, Weimer, Breaker, & Suen, 2019; Subramanian et al., 2014). It is believed that the gut microbiota of animals is established by introducing xenomicrobiota from their surroundings (Backhed et al., 2015; Guzman, Bereza‐Malcolm, De Groef, & Franks, 2015; Koenig et al., 2011), as animals are exposed to complex environments and microbiota since birth (Jin, Wu, Zeng, & Fu, 2017; Ren et al., 2017; Tun et al., 2017). In general, the gut microbiota increases in abundance from birth to adulthood and becomes an adult‐like microbiota with increasing age (Backhed et al., 2015; Faith et al., 2013; Rawls, Mahowald, Ley, & Gordon, 2006; Subramanian et al., 2014; Yassour et al., 2016). In previous studies on ruminants, an increase in the alpha diversity and changes in the predominant bacterial taxa have been observed as animals increase in age (Dias et al., 2018; Dill‐McFarland, Breaker, & Suen, 2017; Jami, Israel, Kotser, & Mizrahi, 2013; Yeoman et al., 2018). Although this notion of a gradual establishment of the gut bacterial community is well accepted, the details of this process remain to be elucidated.

Our study aimed to explore the gradual establishment of gut bacterial ecology in cows from birth to adulthood by analyzing the 16S rRNA gene sequences of microbiota from different gut segments, that is, rumen, abomasum, duodenum, and rectum.

## 2 MATERIALS AND METHODS

### 2.1 Sample collection

Experiments were performed at the Zhong Di breeding stock dairy farm located in Beijing, China. Fifty‐eight healthy female Holstein cattle were enrolled in the study, with ages ranging from 1 week to 5 years old. The cattle were reared under the following conditions and were weaned at 2 months old: 1‐week‐old calves (±3 days; *n* = 12) had free access to milk provided in buckets; 1‐month‐old calves (±3 days; *n* = 8) had free access to milk replacer provided in buckets and a solid starter diet (granules: flaked corn, 3:2); 2‐month‐old calves (±3 days; *n* = 8) had free access to milk replacer provided in buckets, solid starter diet, and hay (50% alfalfa and 50% oats); and 6‐month‐old (±3 days; *n* = 11), 1‐year‐old (±7 days; *n* = 5), 2‐year‐old (±7 days; *n* = 8), and 5‐year‐old (±7 days; *n* = 6) Holstein cows had free access to a total mixed ratio (TMR) consisting of 40% concentrated feed and 60% roughage. Each animal of the same age group was reared in individual pens in the same manner with free access to freshwater for 14 days and then fasted for 12 h before harvest.

The calves and cows were euthanized by intravenous injection of Euthanyl (240 mg/ml; Sigma‐Aldrich, Castle Hill, New South Wales, Australia). Each gastrointestinal compartment (rumen, abomasum, duodenum, and rectum) was isolated with sterile surgical thread to avoid the contents mixing. Digesta samples (2 g) from the rumen, abomasum, duodenum, and rectum were collected. However, due to contamination during sampling, there are no abomasum samples from 1‐week‐old calves. In total, 202 samples were collected from 58 animals. All samples were immediately frozen in liquid nitrogen before being analyzed.

### 2.2 DNA extraction and PCR amplification

Genomic DNA was extracted from 0.5 g of digesta samples using the E.Z.N.A.® Stool DNA Kit (Omega Bio‐Tek, Norcross, GA, U.S.) following the manufacturer's instructions. The quality of the extracted DNA was detected using 1% agarose gel electrophoresis and spectrophotometry (optical density at 260/280 nm ratio). The extracted DNA was stored at −20°C until further analysis. The V3–V4 regions of the 16S rRNA genes were amplified with the universal primers 341F (5′‐CCTAYGGGRBGCASCAG‐3′) and 806R (5′‐GGACTACNNGGGTATCTAAT‐3′). These primers also contained a set of 8‐nucleotide barcode sequences that were unique to each sample. PCRs were performed in triplicate using a 25‐μl mixture containing 2.5 μl of 10× Pyrobest Buffer, 2 μl of 2.5 mM dNTPs, 1 μl of each primer (10 μM), 0.4 U of Pyrobest DNA Polymerase (TaKaRa, Kyoto, Japan), and 15 ng of template DNA. The PCR protocol used was as follows: 95°C for 5 min; 25 cycles at 95°C for 30 s, 55°C for 30 s, and 72°C for 30 s; with a final extension of 72°C for 10 min.

### 2.3 Illumina MiSeq sequencing and data processing

Amplicons were extracted from 2% agarose gels and purified using the AxyPrep DNA Gel Extraction Kit (Axygen Biosciences, Union City, CA, U.S.A) according to the manufacturer's instructions. They were then quantified using QuantiFluor™‐ST (Promega, Madison, WI, U.S.A). The amplicons of the V3–V4 hypervariable regions of the 16S rRNA genes were then sequenced using the Illumina Miseq PE300 sequencing platform (Illumina, Inc., San Diego, CA, U.S.A) by Beijing Allwegene Tech, Ltd (Beijing, China).

Raw sequences were assigned to each sample by barcodes, and low‐quality sequences were then filtered. The following were considered “low‐quality sequences”: (a) raw reads that were shorter than 110 nucleotides; (b) 300‐bp reads that were truncated at any site and received an average quality score <20 over a 50‐bp sliding window, and truncated reads that were shorter than 50 bp; and (c) exact barcode matching, two nucleotide mismatches in primer matching, and reads that contained ambiguous characters. Only clean sequences with an overlap longer than 10 bp were assembled using FLASH‐1.2.11 (Magoc & Salzberg, 2011). Reads that could not be assembled were discarded. Chimera sequences were detected using usearch6.1 (Edgar, 2010). Next, high‐quality sequences were then analyzed using the QIIME pipeline (Caporaso et al., 2010). The representative sequence datasets were classified into operational taxonomic units (OTUs) using a threshold of 97% identity and the UCLUST algorithm (Edgar, 2010). The taxonomy of each 16S rRNA gene sequence was assigned by UCLUST against the GreenGenes database (McDonald et al., 2012).

### 2.4 Maturity of gut microbiota

The maturity index is defined as the similarity between the gut microbiota of a young individual to that of an adult (Subramanian et al., 2014). Here, we assumed that the microbiota in adult cows at 5 years old was fully developed; thus, we could infer that gut microbiota more similar to that of adult cows (5 years old) was more developed. The maturity index was calculated using the modified Bray–Curtis similarity algorithm: Maturity index = $1‐\sum_{i=1}^{n}\left|x_{\mathrm{ij}}‐x_{\mathrm{ik}}\right|/\sum_{i=1}^{n}\left|x_{\mathrm{ij}}+x_{\mathrm{ik}}\right|$, where *x* is the bacterial abundance, *n* is the number of identified OTUs in each gut segment, *i* is the list of OTUs, *j* is the given sample for which the maturity index was measured, and *k* is the sample of an adult cow (5 years old).

### 2.5 Gut microbiota index change fitted to the Gompertz model

The Gompertz model is a nonlinear regression analysis algorithm that is widely used to describe the dynamic changes of S‐shape data, such as growth curves. Chao1, Shannon, and maturity indexes were established by age (weeks) with the Gompertz model: index = *a* * exp(−*b* * exp(−*k* * age)), where “index” is the Chao1, Shannon, or maturity index observed; parameter *a* represents the asymptote of expected top index; parameter *b* is an integration constant related to the index; and parameter *k* is the maturity rate of the Chao1, Shannon, or maturity index.

### 2.6 Data analysis

The diversity matrix was calculated using the QIIME pipeline (Caporaso et al., 2010). A Venn plot was constructed, and hypergeometric tests were performed to present the distribution differences in the core bacterial community using the VennDiagram package (1.6.17); the Bray–Curtis index was calculated using the Vegan package (2.4‐5), and a correlation test was performed with the cor.test function in R software (3.3.0). Comparisons between groups were performed using two‐way ANOVA with the default parameters in the R software (3.3.0), and a *p*‐value <0.05 was considered significant.

## 3 RESULTS

### 3.1 Datasets

Two hundred and two samples (Table A1) were collected in the current study. After sequencing 16S rRNA genes on the Miseq platform and performing quality control, 10,781,199 clean sequences (53,372 ± 33,137 sequences per sample) were obtained. Based on 97% nucleotide sequence similarity, these sequences were assigned to OTUs, which were used to identify different taxonomic levels according to the GreenGenes database. In total, 13,827 OTUs, 508 genera, 174 families, 96 orders, 55 classes, and 28 phyla were identified from this dataset.

### 3.2 Bacterial composition was affected by both gut segment and cow age

**FIGURE 1:**
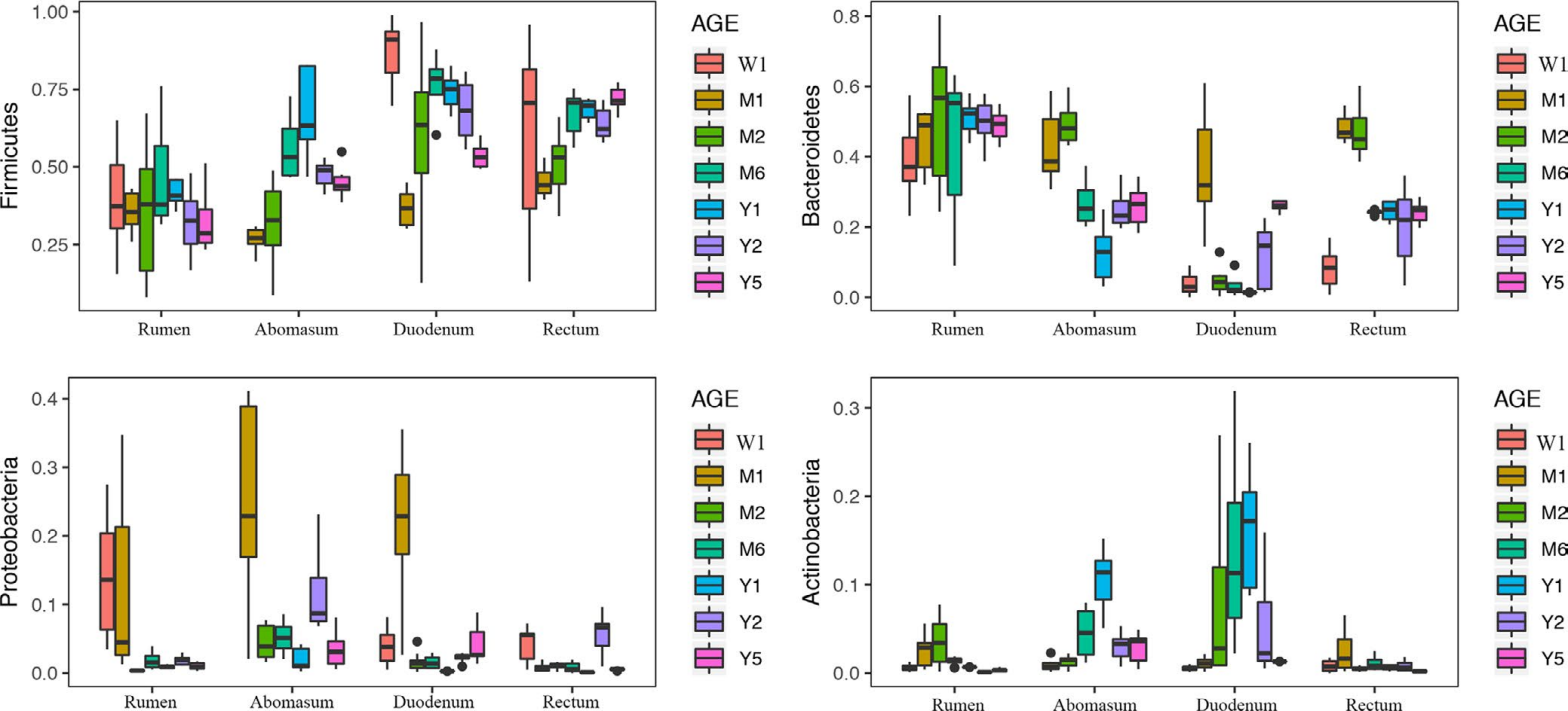
Change in the bacterial composition of predominant phyla in different gut segments in cows with age. The top four phyla with a mean abundance higher than 2.0% are shown (Firmicutes, Bacteroidetes, Proteobacteria, and Actinobacteria). *p*‐value of the fixed effects of age, gut segment, and age × gut segment was lower than 0.05

Based on the established dataset, the majority of the detected sequences in all samples belonged to Firmicutes (~52.14%), Bacteroidetes (~30.72%), Proteobacteria (~6.32%), and Actinobacteria (~3.76%). These four major phyla contributed to almost 93% of total bacteria in terms of abundance, and the relative abundance of these major bacterial taxa was affected by both the gut segment they were found in and the age of the cows (*p* < 0.05, Figure 1).

**FIGURE A1:**
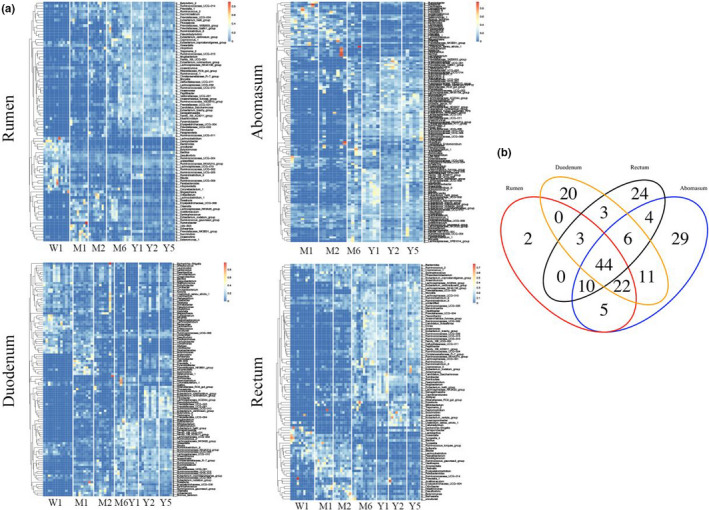
Core bacterial taxa at the genus level in different gut segments. (a) Dynamic change of core genera in the rumen, abomasum, duodenum, and rectum with age increase; the worm color indicated a higher bacterial abundance, while the cold color indicated a lower bacterial abundance. (b) Shared core genera in the rumen, abomasum, duodenum, and rectum (hypergeometric test, *p* < 0.01)

Only 170 of the 13,827 OTUs were identified to species level, and most of these identified species (>98%) were present in less than 50% samples. Thus, genus‐level identification was used to further explore the bacterial taxa changes in different gut segments of cows. Three hundred and fifty‐five genera were identified in the rumen, 329 in the abomasum, 404 in the duodenum, and 268 in the rectum. Among these, 85 genera in the rumen, 131 in the abomasum, 108 in the duodenum, and 93 in the rectum were present in more than 50% of the samples and were considered as the core bacterial taxa for each gut segment. Moreover, the relative abundance of the identified core genera in each gut segment changed as the calves aged (Figure A1a; *p* < 0.05). Furthermore, only a small proportion of the core genera (44/183) were shared across different gut segments, and the distribution of these core genera varied between different gut segments (Figure A1b; hypergeometric test, *p* < 0.05). These observations at the phylum and genus level indicate that bacterial composition may be affected by both the gut segment and the age of the cow.

### 3.3 Bacterial alpha diversity changed with age following a Gompertz curve

**FIGURE 2:**
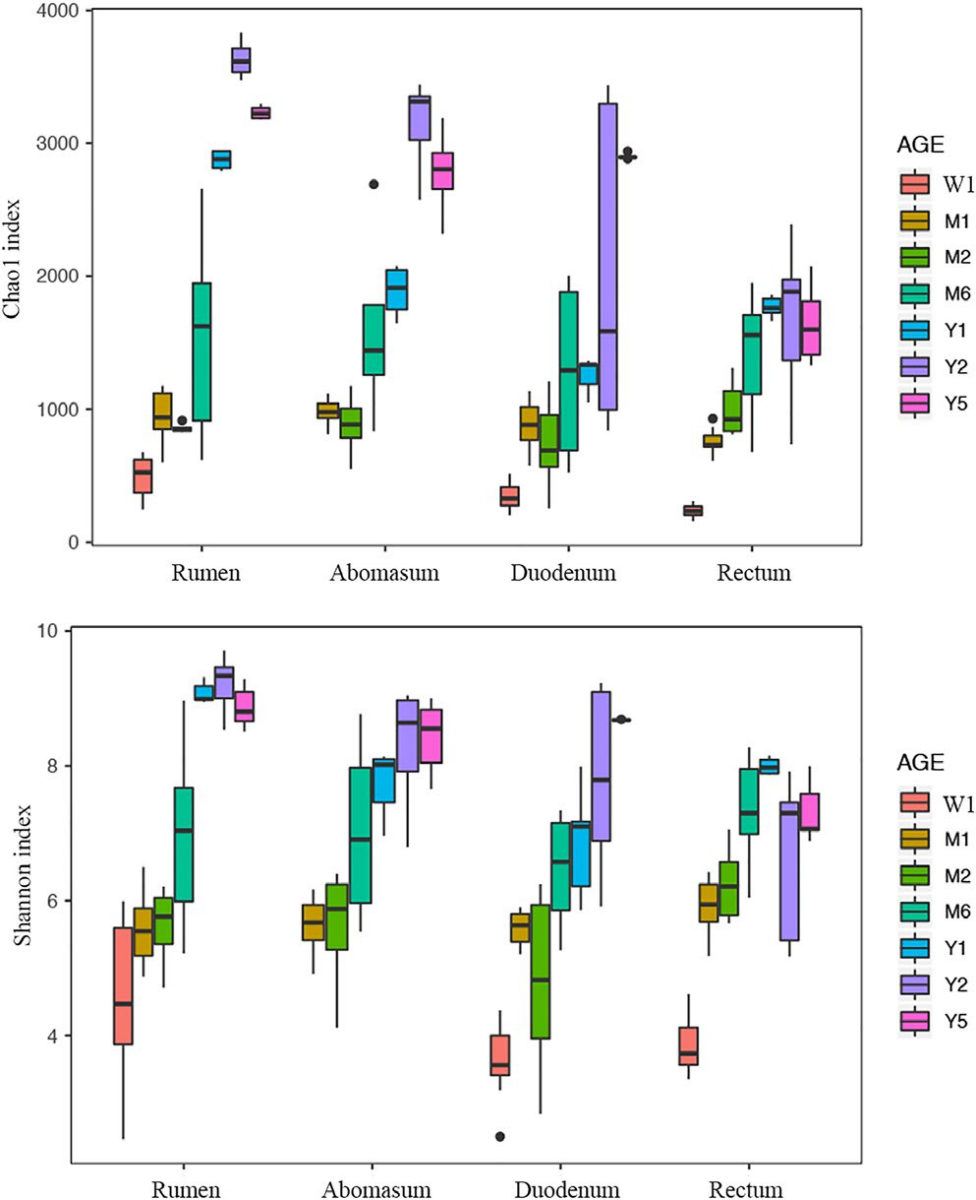
Change of alpha diversity with increasing age in different gut segments of cows. Alpha diversity was calculated via the Chao1 and Shannon indexes. *p*‐value of the fixed effects of age, gut segment, and age × gut segment was lower than 0.05

Both the Chao1 (which reflects the number of expected species) and Shannon indexes (which accounts for both the abundance and evenness of the species presented in a given community), which were used to assess the alpha diversity change, were affected by age (*p* < 0.05) and increased in each gut segment of the cows with age (Figure 2).

**FIGURE A2:**
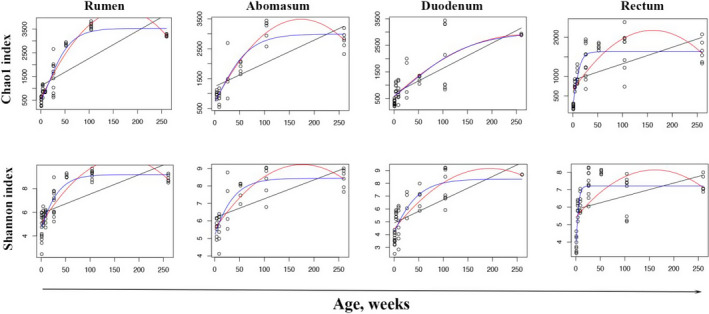
Dynamic changes of Chao1 and Shannon indexes fitted by linear (black), quadratic (red), and Gompertz models (blue)

**FIGURE A3:**
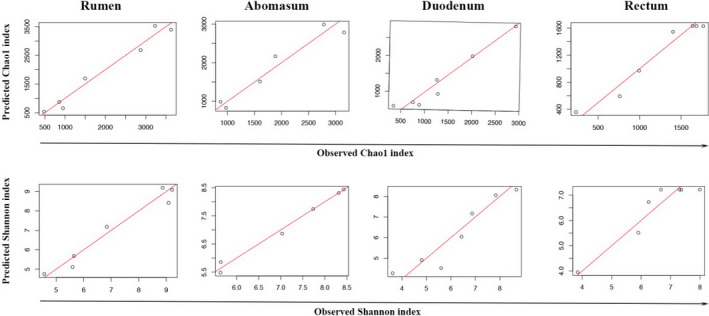
Correlations of predicted and observed Chao1 and Shannon indexes with Pearson correlation

The linear, quadratic, and Gompertz models were used to fit the alpha diversity indexes to the age (in weeks) of the cows. The Gompertz model had the best fit for Chao1 and Shannon indexes for most gut segments based on the Akaike information criterion and Bayesian information criterion (Table A2; Figure A2). The observed indexes and the predicted indexes by the Gompertz model were highly correlated, indicating that the Gompertz model can be used to describe the growth of gut microbiota in the current study (Figure A3; Table A3; *r* > 0.94, *p* < 0.01).

**FIGURE 3:**
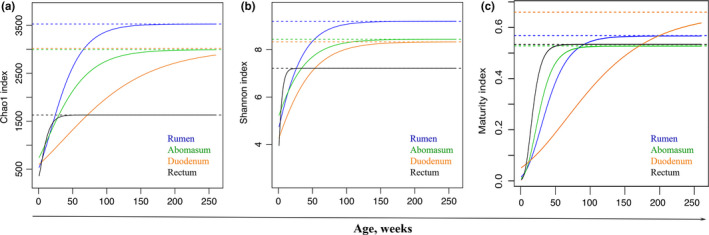
Dynamic change of bacterial alpha diversity and maturity index with increasing age fitted to the Gompertz function. (a) Dynamic change of the Chao1 index. (b) Dynamic change of the Shannon index. (c) Dynamic change of maturity index. Solid lines are the fitted Gompertz curves, and dashed lines are the expected asymptotes. Detailed information is shown in Tables A2, A3, A4, and Figures A2 and A3 for the Chao1 and Shannon indexes, and more detailed information is shown in Tables A5 and A6, and Figure A4 is for the maturity index

By establishing the Gompertz model, the rate at which the bacterial community matured in each bovine gut segment can be found. First, in terms of the mature microbiota of the 4 gut segments, the rumen had the highest alpha diversity (3528 and 9.19 for the Chao1 and Shannon indexes, respectively), followed by the abomasum and duodenum, while the rectum had the lowest alpha diversity (1630 and 7.22 for the Chao1 and Shannon indexes, respectively) (Figure 3a,b). Different growth rates (parameter *k*) of alpha diversity were also found in the gut segments. The microbiota in the rectum matured faster and earlier in life than that in other segments. Based on the Chao1 and Shannon indexes, microbiota in the rectum reached 80% microbiota maturity (close to full maturity) the quickest at 10–20 weeks, followed by the rumen, the abomasum, and the duodenum, which needed substantially more time to reach 80% microbiota maturity (Table A4). The inflection point of the Gompertz model represents the time of the highest microbiota growth rate during maturity, with the inflection of the Chao1 index in the rectum occurring much earlier in life (at 4 weeks old) than in other segments. The inflection point of the Shannon index in each gut segment had a negative value, which indicates that while the Shannon index value infers fast growth early in life, the growth rate gradually decreased with increasing age (Table A4).

### 3.4 Microbiota maturity changed with age following the Gompertz curve

**FIGURE A4:**
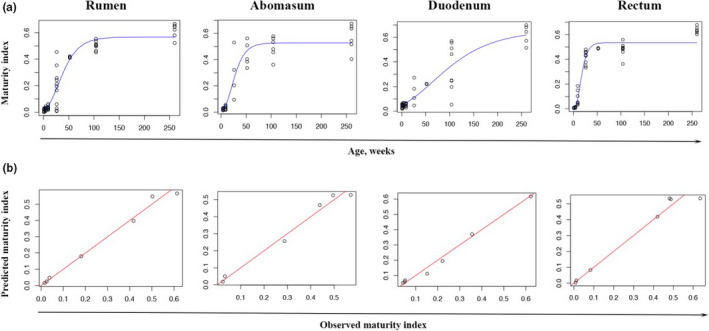
Dynamic change of maturity index fitted by (a) the Gompertz model, and (b) correlations of predicted and observed maturity indexes

The maturity index of microbiota, which was defined as the similarity (Bray–Curtis matrix) between the gut microbiota of young and adult cows (5 years old) at the OTU level, changed in every gut segment as cows aged, following the Gompertz model (Figure A4a). Additionally, the Gompertz model fit the maturity index well because the maturity index predicted by the Gompertz model was highly correlated with the observed maturity index (Figure A4b; Table A5; *r* > 0.98, *p* < 10^−4^).

Under the parameters observed for the Gompertz model of the maturity index, the growth rate of the microbiota maturity index varied between the different gut segments (Figure 3c). Although the parameters of the Gompertz model at maturity were not consistent with those of alpha diversity, a similar trend was observed (Table A6). Microbiota maturity in the rectum was expected to have the fastest growth rate because it had the highest *k* parameter value, followed by the abomasum and the rumen, with the duodenum having the lowest growth rate. The different growth rates were also supported by the predicted age to reach 80% maturity and the inflection age (Table A6). Our findings concerning both the dynamic change of microbiota maturity and alpha diversity indicate that the gastrointestinal bacterial community changed more quickly in younger cows than in older animals. Importantly, the growth rate of the bacterial community in different gut segments also differed, with the highest growth rate found in the rectum and the lowest rate in the duodenum. The bacterial community in the rectum also matured earliest, followed by the rumen, the abomasum, and, lastly, the duodenum.

### 3.5 Dynamic changes of signature taxa in different gut segments

**FIGURE 4:**
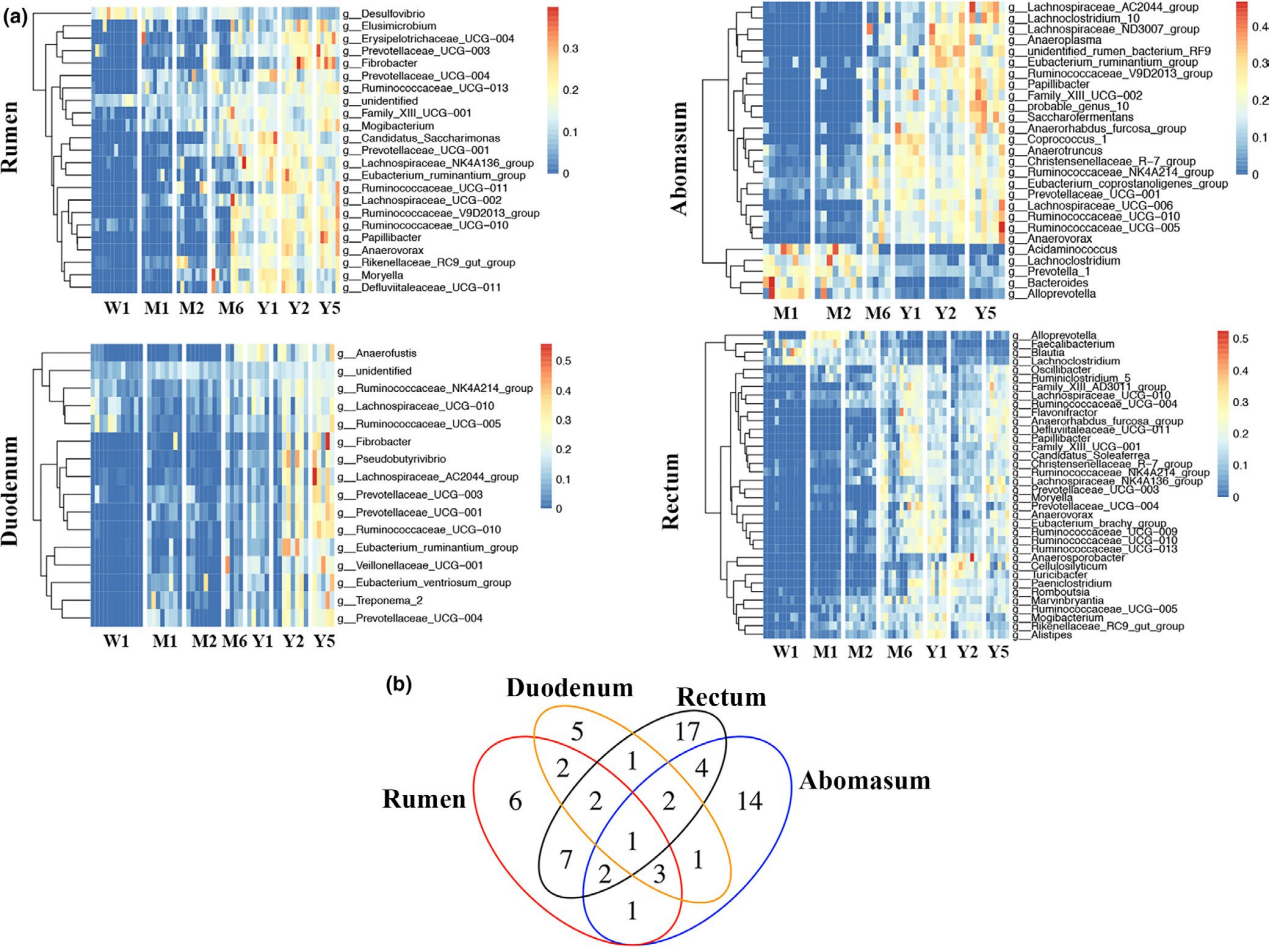
Core bacterial taxa at the genus level that contributed to microbiota maturity in different gut segments. (a) Dynamic change of core genera that contributed to microbiota maturity in the rumen, abomasum, duodenum, and rectum with increasing age (*p* < 0.05). The warmer colors indicate a higher bacterial abundance, while cooler colors indicate a lower bacterial abundance. (b) Shared core genera that contributed to microbiota maturity in the rumen, abomasum, duodenum, and rectum (hypergeometric test, *p* < 0.05)

The relative abundance of the identified core bacterial taxa was fitted to the predicted microbiota maturity in each gut segment to illustrate the dynamic change of bacterial taxa. In the rumen, 22 genera were positively correlated (*r* > 0.43), and 1 genus (*Desulfovibrio*) was negatively correlated (*r* = −0.54) with the predicted microbiota maturity index (*p* < 0.05; Figure 4a). In the abomasum, 22 genera were positively correlated (*r* > 0.53), and 5 genera were negatively correlated (*r* < −0.54) with predicted microbiota maturity index (*p* < 0.05; Figure 4a). In the duodenum, all detected genera (16 genera) were positively correlated (*r* > 0.48) with the predicted microbiota maturity index (*p* < 0.05; Figure 4a). In the rectum, 32 genera were positively correlated (*r* > 0.43), and 4 genera were negatively correlated (*r* < −0.47) with the predicted microbiota maturity index (*p* < 0.05; Figure 4a). Furthermore, the core bacteria which were correlated with microbiota maturity had a little overlap between the different gut segments, with only the relative abundance of *Ruminococcaceae_UCG*‐*010* in all the gut segments fitting the Gompertz model (Figure 4b). These results showed that the bacterial composition in each gut segment dynamically changed as calves aged, and the bacterial taxa that contributed to microbiota maturity also differed between gut segments.

### 3.6 The similarity of bacterial communities in the foregut and hindgut

**FIGURE 5:**
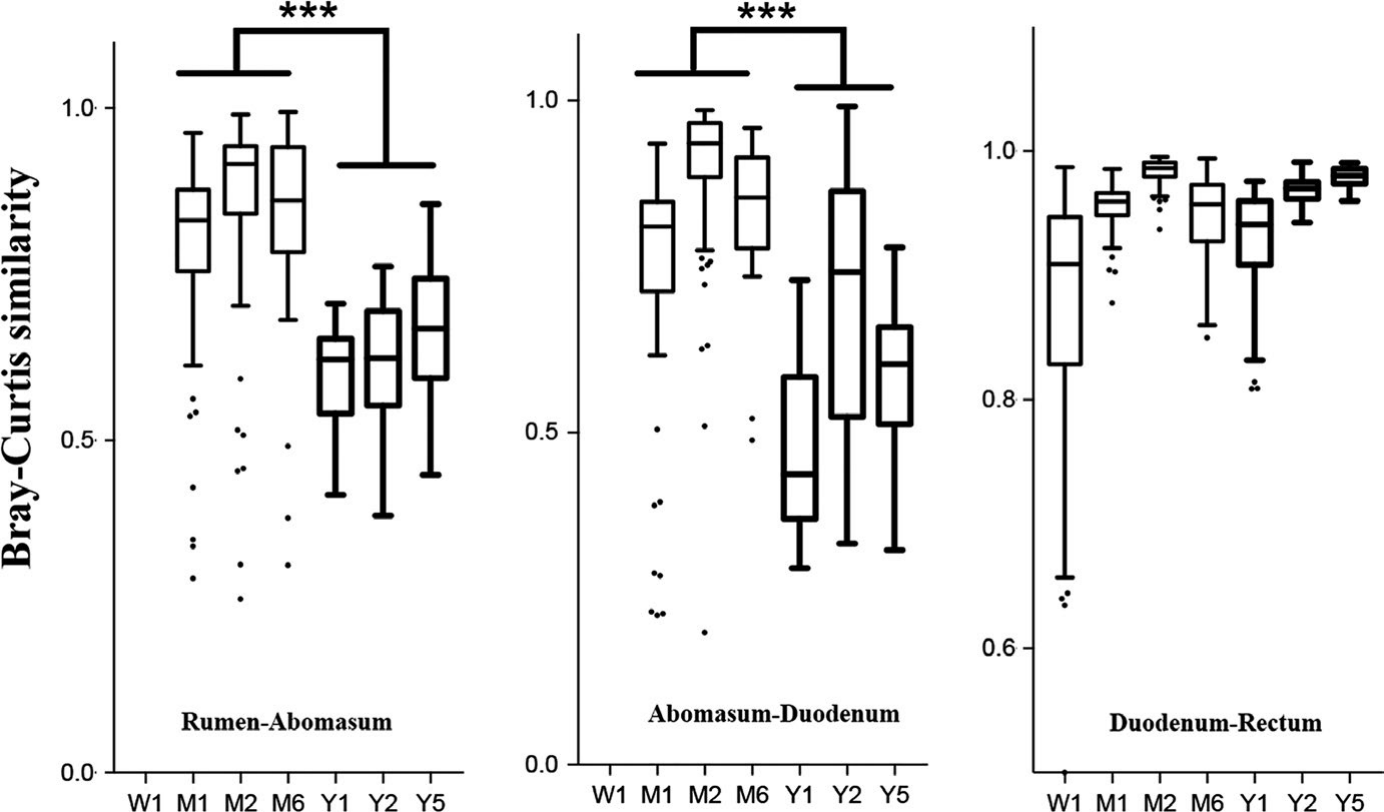
Bray–Curtis similarity of microbiota in the foregut (rumen) and hindgut (rectum) of cows. ***p < 0.05

The bacterial community in the hindgut may be affected by the community in the foregut because of bacterial transfer (Ji et al., 2018). This may explain the similarity between the bacterial compositions in the foregut and hindgut, as active bacterial transfer between the two habitats would result in similar bacterial communities (Jonathan & Robert, 2010). By analyzing the similarity indexes at the OTU level using the Bray–Curtis index, this study found that the bacterial communities in the rumen and abomasum were similar in 1‐, 2‐, and 6‐month‐old calves, but different to those of 1‐, 2‐, and 5‐year‐old cows (*p* < 0.05). Similar results were also observed between bacterial communities in the abomasum and duodenum (*p* < 0.05), although the similarity indexes in the duodenum and rectum were not affected by the cows' ages (*p* > 0.05; Figure 5). These observations of bacterial community similarity and differences in the foregut and hindgut indicate that the similarity in bacterial communities in the foregut and hindgut may be affected by age; with increasing age of a cow, bacterial community similarity in the foregut and hindgut decreased, especially from 6‐month‐old to 1‐year‐old.

## 4 DISCUSSION

The gut microbiota has been demonstrated to be important to the host (Delzenne & Bindels, 2018), especially in ruminants such as cows (Hess et al., 2011; Rubino et al., 2017). Previous studies have shown that bacteria colonized the rumen by the first day after birth, though the rumens of preweaning calves were not well developed (Guzman et al., 2015; Jami et al., 2013; Yeoman et al., 2018), and microbial succession in the rumen and gut then becomes gradually established as cows aged (Dill‐McFarland et al., 2017). However, the process of microbiota establishment was still not fully understood. Therefore, the purpose of the current study was to elucidate this issue from an ecological perspective.

Different gut segments (rumen, abomasum, duodenum, and rectum) in cows were used to explore the establishment of the microbiota because spatial heterogeneity in the microbial community along the gut has been previously demonstrated in mice (Gu et al., 2013; Wang et al., 2010), chickens (Rehman, Vahjen, Awad, & Zentek, 2007), pigs (Looft et al., 2014), humans (Costello et al., 2009), and cows (Godoy‐Vitorino et al., 2011; Yeoman et al., 2018). Although the majority of bacteria in different gut segments belonged to just a few taxa (such as the Firmicutes, Bacteroidetes, Proteobacteria, and Actinobacteria phyla), we observed significant bacterial community differences in terms of alpha diversity, composition, and abundance of dominant and core bacteria between different gut segments, and between cows with different ages. These results are also supported by previous studies in cows (Jami et al., 2013; Li, Connor, Li, Baldwin, & Sparks, 2012) and humans (Koenig et al., 2011; Subramanian et al., 2014; Yatsunenko et al., 2012) and suggest that the relative abundance of bacterial taxa may be affected by both the specific gut segment and the age of the cow. Furthermore, the establishment of bacterial ecology in different gut segments may differ in growth rate (Dias et al., 2018; Yeoman et al., 2018), which can be effectively described from an ecological perspective (Subramanian et al., 2014).

Bacterial alpha diversities, as described by the Chao1 and Shannon indexes, were mostly used to assess the establishment of the gut bacterial community (Guzman et al., 2015; Jami et al., 2013; Yeoman et al., 2018). The maturity index, which also reflects the establishment of the gut bacterial community, is defined as a measure of the similarity between the gut microbiota of calves and that of adult cows (Subramanian et al., 2014). We found that successive gradual change of both alpha diversity and maturity indexes for each gut segment with age followed the Gompertz curve, with gut microbiota established quickly in young animals, then slowing with age. Previous studies detected two pivotal times in the establishment of microbiota in the mammalian gut: The first is at birth when the mammalian gut captures bacteria from the vagina, colostrum, and its surrounding environment to establish its bacterial community (Guzman et al., 2015; Koenig et al., 2011). The second is at weaning, when animals transition from liquid food to solid food, and the gut microbiota shifts quickly to become that of an adult's (Favier, Vaughan, De Vos, & Akkermans, 2002). In humans, the gut microbiota establishes quickly before 6 months of age (Yassour et al., 2016). The gut microbiota of 12‐month‐old infants is similar to those of their mothers (Backhed et al., 2015), whereas the gut microbiota of 3‐year‐olds resembles that of adults (Faith et al., 2013; Yatsunenko et al., 2012). Furthermore, with the parameters of the fitted Gompertz function, we also observed that the growth rates of bacterial communities differed between gut segments, with the highest growth rate found in the rectum. Thus, it can be inferred that the bacterial community in the rectum matured early in life, followed by the rumen and abomasum. The duodenum had the lowest growth rate and matured later than the other segments. The varied growth rate of bacterial communities in different gut segments may be partially ascribed to our findings that show the different signature taxa in each gut segment.

Capturing bacteria from the surroundings (i.e., bacterial transfer) is proposed to play an important role in gut microbiota establishment (Backhed et al., 2015; Guzman et al., 2015; Koenig et al., 2011). Thus, the bacterial community in the hindgut may be affected by the community in the foregut because of bacterial transfer (Ji et al., 2018), which may explain the similar bacterial compositions in the foregut and the hindgut. Work by Jonathan and Robert (2010) demonstrates this, showing that transferring bacteria active between two habitats resulted in similar bacterial communities (Jonathan & Robert, 2010). Here, we demonstrated that the similarity of the bacterial composition shown by the Bray–Curtis matrix between the foregut and the hindgut was high in young animals and decreased sharply with increasing age. This similarity could be roughly dichotomous at around 6 months of age in cows, indicating foregut–hindgut microbiota were more similar up until 6 months, past which point the microbiota composition diverged in cows. These findings imply that the hindgut of younger animals can capture bacteria more easily from the foregut than in older animals, as physical barriers may be established as the gastrointestinal tract develops (Malmuthuge & Guan, 2017), thus preventing microbiota in foregut affecting that in the hindgut. This may also explain why the microbiota in the hindgut matured earlier than the microbiota in the foregut, with the hindgut forming a barrier at an early age.

The limitation of the current study is that a follow‐up design was not used to look at the same individuals over time, which would provide more precise and detailed information. However, in the current study, the microbiota change over increasing age is notable, and the observation of maturity is also comparable with the findings of previous studies on cow rumens (Dill‐McFarland et al., 2017; Jami et al., 2013) and humans (Backhed et al., 2015; Subramanian et al., 2014; Yatsunenko et al., 2012), which can help support our main conclusion.

## 5 CONCLUSIONS

It has been demonstrated that bacterial composition may be affected by both the gut segment they are found in and the age of the cow. This was done by tracking the establishment of the gastrointestinal microbiota in cows from 1 week old to adulthood. Microbiota alpha diversity and maturity changed with age following the Gompertz curve in each gut segment (rumen, abomasum, duodenum, and rectum). The gut microbiota was established quickly in young animals and then slowed with age until it stabilized at maturity. Importantly, differences in Gompertz model parameters were also found in different gut segments, with the highest microbiota growth rate found in the rectum. Thus, we can conclude that the bacterial community in the rectum matured early in life, followed by rumen and abomasum bacteria, with the duodenum having the slowest growth rate and late maturity of the microbiota. The microbiota similarity in adjacent gut segments was higher in younger animals than older animals, which indicates that microbiota in the hindgut may be more easily affected by the foregut microbiota early in life. These findings have expanded our understanding of the dynamic changes of microbiota in different gut segments from birth to adulthood and hints that intervention early in life may be an effective way to manipulate the gut microbiota in cows, thereby improving gut health and the performance of cows.

## CONFLICT OF INTEREST

None declared.

## AUTHOR CONTRIBUTIONS

**Chun Y. Guo:** Investigation (lead); writing – original draft (equal). **Shou K. Ji:** Conceptualization (equal); data curation (lead); formal analysis (lead); writing – original draft (lead). **Hui Yan:** Data curation (supporting); writing – original draft (supporting). **Ya J. Wang:** Writing – review & editing (supporting). **Jing J. Liu:** Investigation (supporting). **Zhi J. Cao:** Writing – review & editing (supporting). **Hong J. Yang:** Methodology (supporting). **Wen J. Zhang:** Conceptualization (equal); methodology (equal); supervision (lead); writing – review & editing (lead). **Sheng L. Li:** Conceptualization (lead); funding acquisition (lead); methodology (lead); supervision (equal); writing – review & editing (equal).

## ETHICS STATEMENT

The experimental design and procedures were approved by the Animal Care and Use Committee of the College of Animal Science and Technology of China Agricultural University (Project number: 31772628) in compliance with the Regulations for the Administration of Affairs Concerning Experimental Animals (The State Science and Technology Commission of P. R. China, 1988).

## Data Availability

Sequence data have been deposited in the NCBI (https://www.ncbi.nlm.nih.gov/sra) under the accession numbers PRJNA649813.

## Appendix A

**TABLE A1:** Numbers of samples collected of different gut sections by cow age

Gut sections	Ages						
	W1	M1	M2	M6	Y1	Y2	Y5
Rumen	12	8	8	11	5	8	6
Abomasum	0	8	8	4	5	6	6
Duodenum	12	8	8	4	5	8	5
Rectum	11	8	8	11	5	8	6

**TABLE A2:** AIC and BIC of Chao1 and Shannon index fitted by linear, quadratic, and Gompertz models

Index	Gut sections	AIC			BIC		
		Linear	Quadratic	Gompertz	Linear	Quadratic	Gompertz
Chao1	Rumen	945	847	853	951	855	861
	Abomasum	588	542	551	592	549	557
	Duodenum	782	778	778	788	786	786
	Rectum	880	852	821	886	860	830
Shannon	Rumen	211	159	150	217	167	158
	Abomasum	112	93	89	118	99	96
	Duodenum	170	152	148	176	159	156
	Rectum	201	191	131	207	199	140

Abbreviations: AIC, Akaike information criterion; BIC, Bayesian information criteria.

**TABLE A3:** Correlations of predicted and observed Chao1 and Shannon indexes

Indexes	Age	Rumen		Abomasum		Duodenum		Rectum	
		Predicted	Observed	Predicted	Observed	Predicted	Observed	Predicted	Observed
Chao1	W1	535	480	–	–	601	343	361	234
	M1	655	949	834	974	641	887	594	760
	M2	875	855	989	873	710	751	970	994
	M6	1694	1497	1516	1602	957	1278	1545	1403
	Y1	2680	2873	2168	1887	1348	1253	1628	1768
	Y2	3394	3627	2783	3156	2029	1997	1631	1686
	Y5	3527	3230	2990	2782	2881	2901	1631	1639
	*r*	0.98		0.96		0.97		0.98	
	*p* value	5.57 * 10^−5^		1.84 * 10^−3^		2.14 * 10^−4^		1.32 * 10^−4^	
Shannon	W1	4.75	4.57	–	–	4.28	3.62	3.96	3.85
	M1	5.11	5.59	5.48	5.63	4.53	5.60	5.51	5.91
	M2	5.68	5.64	5.86	5.64	4.92	4.81	6.73	6.26
	M6	7.18	6.84	6.87	7.03	6.04	6.44	7.21	7.37
	Y1	8.41	9.08	7.74	7.73	7.17	6.87	7.22	7.99
	Y2	9.09	9.21	8.31	8.31	8.06	7.84	7.22	6.67
	Y5	9.19	8.87	8.44	8.43	8.33	8.68	7.22	7.30
	*r*	0.98		0.99		0.94		0.94	
	*p* value	1.22 * 10^−4^		5.75 * 10^−5^		1.36 * 10^−3^		1.67 * 10^−3^	

**TABLE A4:** Gompertz model parameters of the Chao1 and Shannon indexes

Index	Gut sections	Parameters				
		*a*	*b*	*k*	80% maturity (weeks)	Inflection age (weeks)	
Chao1	Rumen	3528	1.96	0.038	72	17.7	
	Abomasum	2992	1.43	0.029	88	12.3	
	Duodenum	3021	1.64	0.014	190	35.3	
	Rectum	1630	1.72	0.133	20	4.1	
Shannon	Rumen	9.19	0.69	0.039	57	–	
	Abomasum	8.44	0.49	0.034	64	–	
	Duodenum	8.33	0.69	0.029	76	–	
	Rectum	7.22	0.79	0.268	10	–	

**TABLE A5:** Correlations of predicted and observed maturity indexes

Age	Rumen		Abomasum		Duodenum		Rectum	
	Predicted	Observed	Predicted	Observed	Predicted	Observed	Predicted	Observed
W1	0.016	0.016	–	–	0.052	0.043	0.004	0.008
M1	0.025	0.025	0.019	0.024	0.058	0.051	0.018	0.012
M2	0.047	0.037	0.050	0.032	0.068	0.052	0.083	0.083
M6	0.180	0.181	0.256	0.288	0.112	0.152	0.418	0.421
Y1	0.398	0.417	0.468	0.439	0.195	0.222	0.528	0.488
Y2	0.548	0.503	0.525	0.495	0.370	0.356	0.534	0.481
Y5	0.567	0.614	0.527	0.571	0.618	0.623	0.534	0.535
*r*	0.994		0.991		0.995		0.982	
*p* value	5.7 * 10^−6^		1.2 * 10^−4^		4.0 * 10^−6^		8.5 * 10^−5^	

**TABLE A6:** Gompertz model parameters of maturity indexes

Gut sections	Parameters				
	*a*	*b*	*k*	80% maturity (weeks)	Inflection age (weeks)
Rumen	0.57	3.73	0.045	65	29.3
Abomasum	0.53	4.38	0.069	50	21.4
Duodenum	0.66	2.58	0.015	176	63.2
Rectum	0.53	5.47	0.120	31	14.1
